# Uncharted routes: exploring the relevance of auxin movement via plasmodesmata

**DOI:** 10.1242/bio.055541

**Published:** 2020-11-12

**Authors:** Andrea Paterlini

**Affiliations:** Sainsbury Laboratory, University of Cambridge, 47 Bateman Street, Cambridge CB2 1 LR, UK

**Keywords:** Plants, Plasmodesmata, Auxin, Cell–cell transport

## Abstract

Auxin is an endogenous small molecule with an incredibly large impact on growth and development in plants. Movement of auxin between cells, due to its negative charge at most physiological pHs, strongly relies on families of active transporters. These proteins import auxin from the extracellular space or export it into the same. Mutations in these components have profound impacts on biological processes. Another transport route available to auxin, once the substance is inside the cell, are plasmodesmata connections. These small channels connect the cytoplasms of neighbouring plant cells and enable flow between them. Interestingly, the biological significance of this latter mode of transport is only recently starting to emerge with examples from roots, hypocotyls and leaves. The existence of two transport systems provides opportunities for reciprocal cross-regulation. Indeed, auxin levels influence proteins controlling plasmodesmata permeability, while cell–cell communication affects auxin biosynthesis and transport. In an evolutionary context, transporter driven cell–cell auxin movement and plasmodesmata seem to have evolved around the same time in the green lineage. This highlights a co-existence from early on and a likely functional specificity of the systems. Exploring more situations where auxin movement via plasmodesmata has relevance for plant growth and development, and clarifying the regulation of such transport, will be key aspects in coming years.

This article has an associated Future Leader to Watch interview with the author of the paper.

## Abstract (precis)

**Summary:** The review discusses the occurrence and biological relevance of auxin transport via plasmodesmata in plants. Physical modes of transport of this hormone, feedbacks on its movement and evolutionary aspects are included.

## INTRODUCTION

“In front of me there were two roads

I chose the less travelled road

And it made all the difference.”

Paulo Coelho – The Witch of Portobello (2006)

Plant growth and development is exquisitely responsive to a range of small, chemically different, endogenous molecules that have received the collective name of plant hormones. These substances can operate as information carriers, act both in proximity and distally from their initial site of biosynthesis and ultimately trigger specific biological responses via signalling or direct action (reviewed in Santner et al., 2009). One of these substances, auxin, is remarkable in the breadth of processes and the range of scales of biological form it has been found to be involved in: from organogenesis (Benková et al., 2003; Reinhardt et al., 2003), overall root (reviewed in Lavenus et al., 2013) and shoot architecture (reviewed in Domagalska and Leyser, 2011) via more local differential growth for tropisms (reviewed in Gilroy, 2008) to fine balances between maintenance of undifferentiated cells (Ding and Friml, 2010), cell division and differentiation (Di Mambro et al., 2017). Auxin is also important for adaptations to abiotic stresses and for interactions with other organisms (reviewed in Kazan, 2013). A comprehensive list would be incredibly long and is one of the reasons why so many plant scientists have had to deal – willingly or not – with this substance, being equally fascinated and challenged by the complexity of its biosynthesis, movement, action and interactions with other regulators. This review will mainly discuss some aspects of the cell–cell movement of auxin. I will employ the word auxin to indicate the main endogenous form of this family of chemicals, indole-3-acetic acid (IAA). However, it is important to remember that other native, biologically active forms also exist and differ in their properties (reviewed in Simon and Petrášek, 2011).

**Fig. 1.**
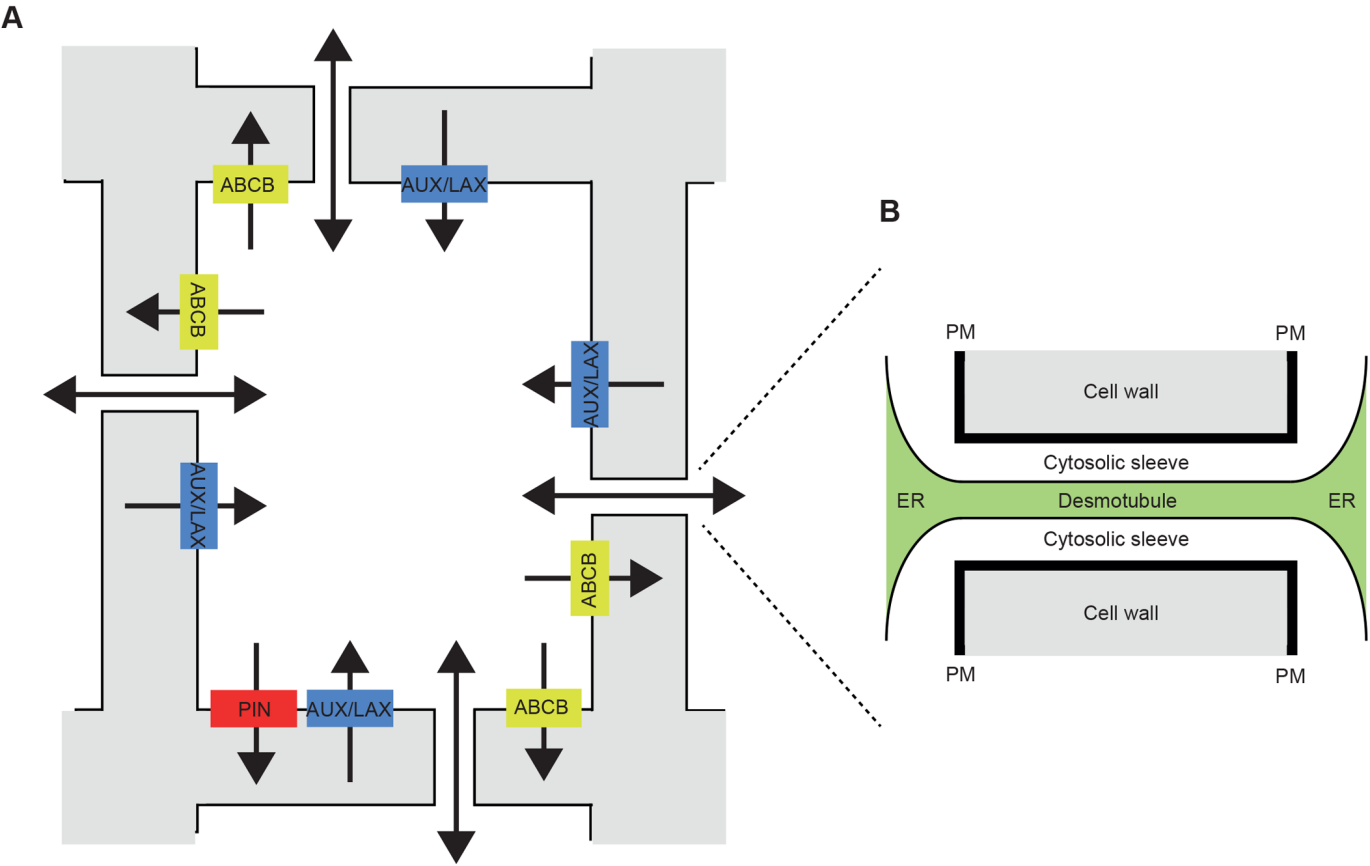
**Auxin transport between plant cells**. (A) Simplified schematic of a plant cell and its immediate neighbours. The cytosol is rendered as white space delimited by a dark line, representing the plasma membrane (PM). The extracellular space (cell wall) is coloured in grey. Transporters of the AUX/LAX family (auxin importers) and those of the PIN and ABCB families (auxin exporters) are depicted on the membrane of the central cell as coloured rectangles. The directionality of auxin transport provided by these proteins is shown as a black arrow. Fluxes across PD are shown as bidirectional arrows as different types of transport could be occurring (see Fig. 2). (B) Zoomed sketch of a PD also showing the endoplasmic reticulum (ER) transversing the channel, the desmotubule, and the cytosolic sleeves.

In chemical terms, auxin is a weak organic acid, so at the mildly acidic pHs of the extracellular space some of the molecules would remain protonated, while others would become negatively charged (Rubery and Sheldrake, 1974; Raven, 1975). While the formers can freely cross the membrane bilayer, active transporters of the AUXIN1/LIKE AUX1 (AUX/LAX) family enable cellular influx of the latter species (Bennett et al., 1996; Yang et al., 2006; Péret et al., 2012). Once inside, at cytosolic pH, auxin would be almost entirely in the deprotonated form (Rubery and Sheldrake, 1974; Raven, 1975). Efflux from the cells therefore has to be mediated by members of the PIN-FORMED (PIN) family (Gälweiler et al., 1998; Petrášek et al., 2006; Bennett et al., 2014a) or the ATP binding cassette B (ABCB) family (Noh et al., 2001; Terasaka et al., 2005) of active transporters. Polar localisation of the transporters on the membrane provides directionality to the auxin fluxes within tissues (Wiśniewska et al., 2006). This is especially the case for PINs while AUX/LAX and ABCBs display this to lower and more cell-type-dependent degrees, being in general more uniformly distributed in the membranes of cells (Gälweiler et al., 1998; Swarup et al., 2001; Geisler et al., 2005) (Fig. 1).

**Fig. 2.**
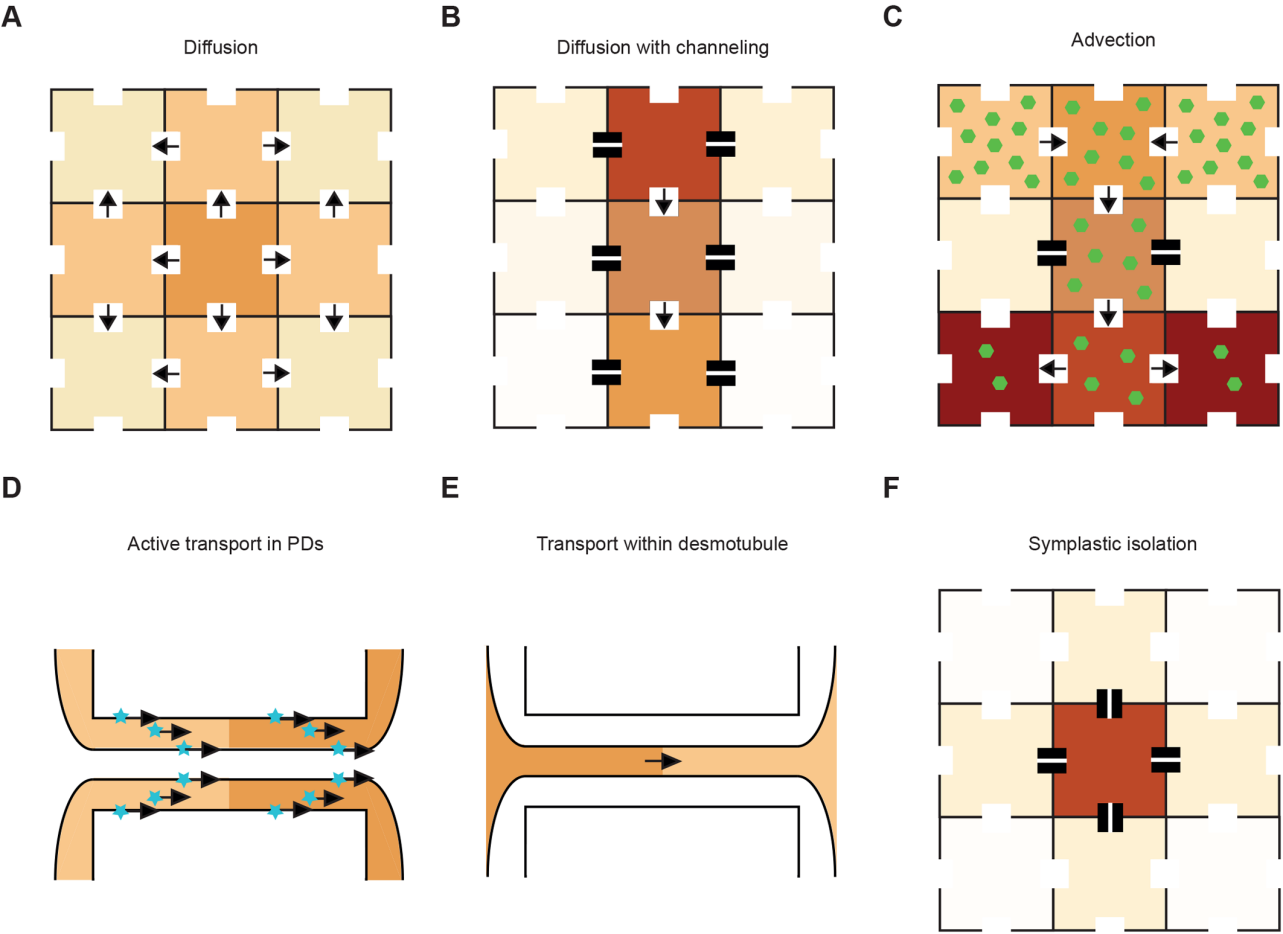
**Potential modes of auxin transport across PD**. (A–F) various proven, or potential, auxin transport mechanisms across PD. Cells are depicted as neighbouring tiles with dark contours. The colour of the squares is proportional to the intracellular auxin concentration, darker colours signifying higher amounts. PD are rendered as white gaps in the edges of the cells. Arrows show symplastic auxin fluxes between cells. Callose accumulation is depicted as two black rectangles. Metabolites (or other substances generating a drive for bulk transport) are shown as green hexagons. The number of hexagons is proportional to the intracellular concentration of the hypothetical metabolite. Zoomed views of PD depict a central desmotubule, surrounded by the cytosolic sleeve and delimited (above and below) by the plasma membrane of the cell. Potential factors interacting with auxin for its active transport (cytosolic or embedded in membranes) are shown as light blue stars. These factors, the metabolites and callose are not shown to scale. Transporter driven auxin fluxes would also be present in the cells represented (and could alter the patterns displayed) but are not considered for simplicity of representation.

Other signalling substances and metabolites move between cells without the need for transporters and without having to leave the cellular symplastic space. Plasmodesmata (PD), membrane lined channels spanning the walls of neighbouring cells, enable this transport (reviewed in Li et al., 2020) (Fig. 1). Movement is largely governed by the size and shape of the substance, which has to be compatible with the aperture of the channel (Terry and Robards, 1987). The auxin molecule falls within such a category (Han et al., 2014; Rutschow et al., 2011) so transport is, and has always been, more likely to occur than not. Some authors did indeed consider this transport component very early in auxin research (Mitchison, 1980; Arisz, 1969, for example).

The argument around auxin movement via PD is whether this transport has *biological significance.* Namely, if it appreciably influences concentrations and distributions of the hormone in tissues and if it contributes to the developmental processes depending on such parameters. The alternative vision, perhaps implied until the recent developments discussed in this review, regards auxin PD movement as some form of non-functional leakage that actually creates issues for active auxin transport (Rutschow et al., 2011). I will show here that there is sufficient evidence to discard this second hypothesis and more attention should be given to auxin symplastic transport.

It is also important to consider that mutations in auxin transporters, while leading to phenotypes at times even severe (see for instance *pin1* in Gälweiler et al., 1998), are not lethal (sextuple *pin* mutants in Verna et al., 2019). The lines without such proteins were instead instrumental in generating a breadth of literature on the relevance of active auxin transport. Mutations removing PD, conversely, have never been identified, and this will most likely continue to be the case. Even mutations altering PD permeability can be embryo lethal (Kim et al., 2002). A slight bias in considering the biological significance of auxin transport mechanisms might therefore have arisen from that.

This is not to challenge the centrality of transporter-driven auxin movement: this route remains an essential one especially in processes requiring transport up concentration gradients to generate auxin maxima or those involving sharp auxin gradients (Heisler et al., 2005; Benková et al., 2003, as examples). The transporters also provide an extremely refined and tuneable directionality system at the cellular scale (Zhang et al., 2020; Huang et al., 2010, as examples). PD transport could instead integrate into this picture and explain situations where transporters alone do not seem sufficient to explain the biology of the system (Mellor et al., 2020; Verna et al., 2019; Guenot et al., 2012, as potential examples).

In this review, I will highlight the various types of movement auxin could experience across PD. I will then link those to biological situations where auxin PD transport has been shown to, or could, contribute to developmental processes. I will then raise points on potential integration of transporter and PD-driven transport mechanisms, including feedbacks that have been observed between the two. I will conclude with a brief consideration on the evolutionary history of PD and the PIN family of auxin transporters.

## TYPES OF MOVEMENT AUXIN MIGHT EXPERIENCE ACROSS PD AND THEIR BIOLOGICAL RELEVANCE

### Diffusion

Diffusion down concentration gradients existing between cells is perhaps the simplest type of movement that can occur across PD (Schönknecht et al., 2008) (Fig. 2A). Diffusion of soluble molecules would primarily occur in the cytosolic sleeve of PD (also called cytoplasmic sleeve in the literature) (Fig. 1B). In the context of auxin, this was recently highlighted in Mellor et al. (2020). The authors, focusing on the root tip of Arabidopsis, compared the observed signal intensity of a DII-VENUS reporter (where fluorescence negatively correlates with auxin concentration) (Brunoud et al., 2012) with that predicted by a modified computational model of them (Band et al., 2014). In this version both auxin active transport and some local biosynthesis were included. Nonetheless, much sharper differences between high and low auxin areas were predicted than those that were observed. This held true even when extensive parameter space was surveyed and in several genetic backgrounds, namely mutants for active transporters. Transport via PD was incorporated into the model and the authors then tested whether such a route contributed to the functional auxin distribution within root tips. Symplastic auxin movement indeed reduced the sharpness of concentration differences and provided better agreement with those observed experimentally. Specifically, this form of movement was essential to facilitate reflux from the high auxin areas in the outer tissues, where active transporters direct flow shoot-ward, to the inner central tissues where flow is root-ward and the auxin concentration is lower (Mellor et al., 2020). This reflux component was theorised in Grieneisen et al., 2007 and is necessary to retain high auxin concentrations in the root tip, something necessary for many biological processes.

Increasing PD permeabilities influenced auxin dependent DII-VENUS signals in general agreement with model predictions (Mellor et al., 2020). Changes were achieved both via chemical treatments (Rutschow et al., 2011) or using inducible antisense lines against a callose synthase gene (GSL8), whose polysaccharide product lines PD and regulates their aperture (Han et al., 2014). Differentials between observed and predicted values were, however, larger here, likely due to necessary approximations in the predicted effects of these manipulations on PD permeability.

When considering auxin movement across PD, attention should also be given to the negatively charged nature of this substance at cytosolic pHs (Rubery and Sheldrake, 1974; Raven, 1975). Electrostatic interactions with PD constituents could be occurring. The lipids on the inner leaflet of the plasma membranes (PM) lining PD are strongly negatively charged (Simon et al., 2016). Research regarding the importance of electrostatic charges of molecules crossing PD is limited (Terry and Robards, 1987, as an example). One paper did not find robust support for the idea that charge contributed significantly to the ease of movement of green fluorescent protein variants (Dashevskaya et al., 2008).

### Diffusion with channelling effects

Passive – as opposed to energised – transport is not necessarily synonymous with unregulated transport. Movement across PD is indeed tightly and finely controlled (reviewed in Li et al., 2020). While still obeying movement down concentration gradients, the specific available space for passive transport could be restricted. As a result, transport would be granted a form of directionality (Fig. 2B).

An example of this, in the context of auxin, was recently provided in Gao et al. (2020). The authors quantified cell–cell coupling in leaf tissues using a photoactivatable dye (Martens et al., 2004). Movement, from the activated cell, was asymmetrical and biased in the longitudinal direction in midrib and petiole epidermal cells. The same was also observed, albeit to a lower extent, in deeper underlying tissues but not in leaf pavement cells to the sides of the midrib. As higher levels of callose were detected on the transverse walls, channelling of transport in the longitudinal direction might be achieved by altering the permeability of PD themselves. While the authors did not clarify how such selective callose deposition could be enforced, they showed that permeability differences were abolished in a *gls8*-callose synthase mutant. Using a radiolabeled version of auxin, applied to the leaf tip, more auxin arrived (sooner) in the petiole of wild-type plants compared to that of the mutant. Channelling might aid movement, possibly by reducing dissipation (Gao et al., 2020).

The paper also provides functional relevance for this symplastic auxin flow: the callose mutant displays reduced leaf hyponasty (leaf petiole bending) upon auxin application to the leaf tip (Gao et al., 2020). Hyponasty depends – among other processes – on auxin flow from the tip of the leaf into the petiole (van Zanten et al., 2009; Michaud et al., 2017; Pantazopoulou et al., 2017). The auxin active transporters involved in hyponasty did not display altered transcription in the callose mutant (Gao et al., 2020), so the effect might be largely attributable to PD transport. PIN proteins experience extensive post-translational modifications (reviewed in Löfke et al., 2013) so a contribution of that can't be ruled out, albeit how that could be connected to callose levels seems hard to envisage. The effect is not absolute, as some upward bending still occurs, nor does it obviously affect the fitness of the mutant allele employed, from what visible in the picture. However, testing the genotype in a crowded setting, upon flooding or thermal challenges would be relevant as the hyponastic responses displayed in those conditions (Millenaar et al., 2005) could be sharply relevant for plant fitness (reviewed in van Zanten et al., 2010).

Interestingly, the different PD densities across the various cell types of the root tip (Zhu et al., 1998) seemed determinant for the symplastic auxin fluxes described in Mellor et al. (2020). Modelled uniform densities were unable to recapitulate DII-VENUS patterns. PD distributions might therefore provide a quantitative form of directionality even in diffusive processes.

Channelling effects via callose deposition might not be restricted to leaves or the epidermal layer. For instance, PD in proto sieve elements higher up than those performing phloem unloading appear occluded by callose (Ross-Elliott et al., 2017) and the meta sieve element-companion cell complex is largely symplastically isolated from surrounding tissues in the root (Oparka et al., 1994; Knoblauch et al., 2015). These features could support long distance basipetal channelling of auxin within the phloem. Transport of the hormone in this tissue, in relation to bulk flow mechanisms, is described in the next section.

### Advection

Another form of passive motion is advection, which occurs when the bulk flow of another fluid carries a substance along. Such movement would carry an overall directionality and, in addition, it could achieve movement of auxin up concentration gradients (Fig. 2C). Please note that while advective transport would be passive, an active mechanism might be required to generate the drive for bulk motion. Long distance transport in the phloem tissue is most likely based on bulk flow: osmotically generated pressure pushes the fluid towards areas of lower solute concentration (Knoblauch et al., 2016).

All steps of phloem function; loading, translocation and release of transported substances are dependent on PD, or modified forms of the same, connecting the relevant vascular cells (Ross-Elliott et al., 2017; Dettmer et al., 2014; Rennie and Turgeon, 2009). Advection is therefore an extremely relevant form of transport across PD.

Presence of radiolabelled auxin in the phloem, when the tracer is specifically applied to mature leaves, has been reported in a range of species and shown to be unaffected by inhibitors for active auxin transport (Morris and Thomas, 1978; Morris et al., 1973). Significant amounts of endogenous IAA have also been detected in phloem sap (Allen and Baker, 1980). Symplastic movement within phloem was clearly shown in Bishopp et al., 2011, when root-detected radiolabelled auxin was diminished upon phloem connectivity impairment. Phloem PD, and possibly sieve pores, were specifically occluded via induced callose deposition (Vatén et al., 2011). Potential feedback on active transport in response to blocked symplastic trafficking were not extensively assessed, but PIN7 displayed a slight reduction in its domain of expression in the root (Bishopp et al., 2011).

Active and passive transport of auxin in relation to the phloem might cooperate. While an active transport inhibitor did not affect the phloem translocation of the hormone, it did cause a reduction of its uptake in leaf veins (Goldsmith et al., 1974). In a mutant for *AUX1*, a reduction in an auxin reporter was observed in leaf vasculature (Marchant et al., 2002) and roots (Swarup et al., 2001). The authors speculated that the transporter might help load/unload auxin into/from the phloem. The transporter was expressed in protophloem of the root (Swarup et al., 2001) but was not specifically restricted to leaf vascular tissues (Marchant et al., 2002). The ABCB19 transporter has been suggested, conversely, to retain auxin in the phloem system along the transport pathway (Blakeslee et al., 2007). Shoot to root IAA transport was strongly reduced in the mutant (Noh et al., 2001) and the transporter displayed expression in the pericycle and endodermis, although present more broadly (Blakeslee et al., 2007).

However, especially in the context of advection, the presence of auxin does not immediately equate to functional relevance. In Bishopp et al. (2011) the impairment of symplastic phloem connectivity, and the associated reduced auxin transport, did not lead to large changes in root auxin responses. The changes observed were actually attributable to delivery of another hormone, cytokinins. The phloem is therefore unlikely to be a biologically *necessary* route, at least for the general signalling processes tested in that paper. Alternatively, compensation mechanisms might buffer reductions in this symplastic transport. Along similar lines, shoot-derived auxin (travelling via any potential route) was insufficient to maintain root meristem identity when local biosynthesis was compromised (Brumos et al., 2018).

This does not altogether rule out a significance for phloem auxin. Temporal differences might exist: lateral root emergence, when seedlings are young, was shown to depend on shoot-derived auxin (Bhalerao et al., 2002). Environmental ones are also possible, since increased lateral root development under high humidity conditions was attributed to higher auxin phloem transport (Chhun et al., 2007). Transport of auxin in the phloem would have a clear speed advantage, around one order of magnitude, to that via active transporters (Sabnis and Watson, 1982; Tsurumi and Wada, 1980; Kramer et al., 2011). It might therefore carry functional relevance in specific situations where fast communication is required or advantageous.

Spatial aspects might similarly apply. For instance, one can envisage a relevance for phloem movement of auxin within the transport pathway itself rather than at the terminal release point. Auxin is well known to be involved in vascular development (see Sachs, 1981, for a classic example). It is interesting that a callose mutant in maize, *tie-died2*, impaired in loading of molecules from companion cells to sieve elements, also presents vascular developmental defects (Slewinski et al., 2012). Movement of protein regulators (as speculated in Baker et al., 2013) but also perhaps auxin might be affected. PD auxin movement was recently speculated, in the context of leaf vein patterning, in Ravichandran et al., 2020.

The movement in the phloem of auxin-signalling components influencing responses to this hormone at sites of unloading has also been reported (Notaguchi et al., 2012; Spiegelman et al., 2015), but is not the specific focus of this review.

Combinations of types of transport are also possible, for instance phloem unloading in Arabidopsis is convective, combining both diffusion and advection (Ross-Elliott et al., 2017).

### Active symplastic transport

Auxin transport across PD does not need to be (or always be) passive in nature. Energy-dependent movement could be relevant and could even enable transport against concentration gradients. While non-targeted transport across PD largely relies on structural properties of the substance, targeted movement involves the modification of PD permeability by the substance to be transported (or associated partners) to facilitate its own movement (Crawford and Zambryski, 2000) (Fig. 2D).

Directional yet non-targeted transport across PD in moss protonema (Kitagawa and Fujita, 2013) and in trichomes (Christensen et al., 2009) was also shown to rely on energy in some capacity, as it was abolished upon metabolic inhibition. The fact that directionality was not absolute (Kitagawa and Fujita, 2013; Christensen et al., 2009; Howell et al., 2020) and that the phenomenon was observed in both non-secreting and secreting trichomes (Christensen et al., 2009) makes the process less likely to be due to bulk flow. Overall, the lack of precise data (also in relation to auxin) would warrant further research into this type of directional transport via PD.

### Diffusive transport within the desmotubule

So far, I have considered (auxin) movement across PD only within the cytosolic sleeve. This is the space between the plasma membrane lining these channels and a constricted form of the endoplasmic reticulum (ER) – the desmotubule – that also runs through PD (reviewed in Li et al. (2020)) (Fig. 1B). While the sleeve is likely the main route for transport of hydrophilic substances, diffusive movement within the constricted ER lumen has been shown for small dyes (Barton et al., 2011; Cantrill et al., 1999). Auxin size could be compatible with such transport (Fig. 2E).

In this regard, it is interesting that active auxin transporters are localised to the ER and are involved in import of the hormone into this cellular domain. These proteins include some PINs with specific structural features (Mravec et al., 2009; Ding et al., 2012) and the family of PIN-likes (PILS) proteins (Barbez et al., 2012). While ER import is regarded as a sequestration mechanism to determine cellular sensitivity to auxin (reviewed in Barbez and Kleine-Vehn, 2013), loading of auxin into the ER for cell-cell transport can't be ruled out.

### Restricted symplastic transport

The presence of PD could be problematic for biological processes where auxin maxima are locally generated or gradients are enforced via active transport. Open PD could partially dissipate such auxin levels by leakage into surrounding cell layers. Preventing symplastic trafficking of auxin might be convenient in such scenarios and can be envisaged – with some flexibility on the term transport – as a form of highly restricted and regulated movement (Fig. 2F).

Auxin plays a central role in all stages of lateral root development: priming the future branching site, influencing division patterns and helping root emergence through overlying tissues. These processes are achieved via combinations of auxin maxima, gradients and overall signalling (reviewed in Lavenus et al., 2013). Symplastic connectivity of the lateral root primordium (LRP) and surrounding tissues also plays key roles in lateral root development (Benitez-Alfonso et al., 2013; Sager et al., 2020). Specifically, LRPs become fully isolated via callose accumulation around stages IV–V (Benitez-Alfonso et al., 2013). It is fascinating to speculate that this might be important to preserve the sharp auxin gradient (decreasing from the tip of primordium to its basal sides) observed using a DR5 auxin-signalling reporter at those stages (Benková et al., 2003). The gradient was dependent on active transport as more diffuse signals were observed upon treatments with transporter inhibitors or auxin analogues that are poorly taken up by transporters (Benková et al., 2003). It is less likely that LRP isolation has the goal to overall retain auxin, as much stronger DR5 signals are observed at earlier stages (Benková et al., 2003) when symplastic connectivity is still present (Benitez-Alfonso et al., 2013).

Active accumulation of auxin is also necessary for tropic responses of the hypocotyl. High auxin accumulates on the side that will go on to display increased growth and bending (Friml et al., 2002 as an example). However, GSL8-dependent accumulation of callose on that side was also observed during photo/gravitropisms (Han et al., 2014). Interestingly, in an inducible knockdown of *GSL8* or upon treatments with callose synthesis inhibitors, the curvature responses were abolished. Higher transport of radiolabelled auxin and broader signals for auxin reporters were observed in the hypocotyls in the knockdown line. The normal auxin gradient across the hypocotyl was also specifically affected. Inhibitors of active transport did not modulate these aspects, suggesting that the phenotypes were most likely the result of increased auxin movement via PD (Han et al., 2014). PIN3 localisation was not affected (Han et al., 2014) but PIN4 and PIN7, equally important, were not checked. Callose accumulation might therefore be necessary to restrict high auxin concentrations to the side of the hypocotyl that will display tropic bending.

It is important to consider that symplastic isolation might have trade offs with delivery of metabolites to cells. Fine regulation of these processes is therefore likely required.

## RECIPROCAL FEEDBACKS BETWEEN AUXIN AND PLASMODESMATA

### Auxin modulating PD permeability

In a less specific way compared to targeted transport, auxin could generally regulate PD permeability and increase or decrease the movement of many substances (among which itself). For instance, in the context of the hypocotyl phototropic and gravitropic responses I described, auxin signalling directly upregulates transcription of the callose synthase *GSL8*, shown to be important for those processes. A positive feedback loop is generated: auxin promotes its own accumulation on one side of hypocotyl by blocking symplastic communication and diffusion (Han et al., 2014).

Additional examples come from the study of lateral root development. Expression of Plasmodesmata callose binding (*PDCB*) protein 1, involved in callose stabilisation and possibly deposition (Simpson et al., 2009), was upregulated by auxin signalling (Parizot et al., 2010; Maule et al., 2013). Reporters for the gene showed signal in LRP from stage III to VI (Maule et al., 2013). It is therefore likely that PDCB1 contributes to the LRP symplastic isolation at stages IV–VI. In this regard, it would be attractive to study phenotypes in loss-of-function mutants and see the impact on auxin fluxes.

However, overexpression of *PDCB1* resulted in higher density of LRPs (Benitez-Alfonso et al., 2013), something not immediately relatable to their symplastic isolation at later stages. As the use of a constitutive promoter would cause ectopic callose accumulation early in the process, priming of future LRPs might have been affected. Clusters of high DR5 signal in xylem pole pericycle cells (the future branching sites) were indeed observed and later resulted in closely positioned, rather than orderly spaced, roots. The same phenotype was observed when callose was induced from a specific xylem pole promoter (Benitez-Alfonso et al., 2013). Cell–cell isolation likely disrupts the initial symplastic phase of LRPs. A speculative interpretation is that, at those stages, an initial active transport-driven auxin influx needs to be quickly dissipated for the signalling to be limited to a few founder cells (Casimiro et al., 2001). Sustained levels of auxin, because of impaired drainage, could lead to wider areas of priming. Movement of inhibitory regulators out of primed cells into surrounding ones is equally possible.

The LRP spacing phenotype also appeared in mutants for PD beta glucanases (*PdBG*) 1 and 2 (Benitez-Alfonso et al., 2013), which are involved in callose degradation (Levy et al., 2007; Iglesias and Meins, 2000). *PdBGs* are expressed in xylem pole pericycle cells and are also similarly induced by auxin signalling (Parizot et al., 2010; Benitez-Alfonso et al., 2013). However, as their strongest expression was at stage III, just before symplastic isolation, a specific function then is harder to envisage. Draining of the primordium of auxin or other signals might be necessary before isolation.

Another form of auxin regulation of PD occurs during the emergence of lateral roots. It was shown that ectopic induction of *PDCB1* resulted in reduced emergence, likely because of callose induction in tissues overlying LRPs (Maule et al., 2013). This was confirmed in Sager et al. (2020) by overexpressing another protein, PD localised protein (*PDLP*) 5. When the native domain of expression of *PDLP5* was studied, the authors observed that signal occurred in cell layers above LRPs and accompanied lateral root emergence. *PDLP5* was also induced by auxin signalling. Connections with the movement of auxin itself were suggested: in the *pdlp5* mutant (likely presenting more permeable PD), a higher number of DR5- and LAX3-expressing cells were observed in the layers above LRPs. LAX3 is normally involved in auxin influx from LRP into those overlying cells (Swarup et al., 2008). Higher auxin levels might therefore be present in those cells in the mutant. Considering the positive role of auxin in altering cell wall components to facilitate passage of the new root across the tissues of the primary one (reviewed in Péret et al., 2009), this well agrees with the observed increased lateral root emergence in the mutant (Sager et al., 2020).

Auxin can increase the activity of pectin methyl transferase proteins and modify the pectin component of the cell wall (Bryan and Newcomb, 1954; Laskowski et al., 2006; Braybrook and Peaucelle, 2013). Interestingly, these polysaccharides are enriched at PD (visible in Faulkner et al., 2008) and members of the pectin methyl transferase protein family have been localised to PD (Morvan et al., 1998). One member was also shown to interact with viral components and facilitate their spread, potentially by altering PD (Chen et al., 2000). Auxin might, therefore, not only facilitate separation of cells overlying lateral root primordia but also reinforce its own movement to achieve such effect.

However, the exact role played by PDLP5 in normal conditions remains puzzling: what selective advantage is provided by the induction of a protein negatively regulating lateral roots emergence, when the inducer itself favours it? Would this provide some control over lateral root numbers or would this once again serve to give full control to active auxin transport mechanisms in those cell layers? It will be interesting to see a future coherent framework integrating auxin processes and symplastic connectivity in lateral root development.

It is important to remember, overall, that since auxin treatment did not increase cell–cell movement of fluorescent dyes in the root (Rutschow et al., 2011), tissue or temporal differences must also exist in these types of feedbacks.

### Symplastic connectivity altering auxin biosynthesis and active transport

Feedbacks on auxin processes can also occur in response to altered cell–cell communication. An example of this was observed when cell-type-specific accumulation of callose was induced within the quiescent centre (Liu et al., 2017). This cellular domain acts as an organiser centre for the root meristem (Sarkar et al., 2007). In addition to loss of stem cell maintenance, the symplastic isolation also resulted in auxin reduction in the proximity of the quiescent centre. The auxin gradient normally present along the root cap was also disrupted (Liu et al., 2017). Auxin levels are fundamental for stem cell maintenance in the root (Ding and Friml, 2010). Interestingly, several auxin biosynthesis genes (but not active transporter genes) displayed reduced expression in the treated roots. PIN protein localisation was also largely unaltered, further suggesting that the observed auxin changes were largely due to biosynthesis (Liu et al., 2017). Movement of unknown regulators via PD might therefore instruct the formation of auxin gradients within the root tip. A symplastic auxin component diffusing from the QC might also contribute.

The callose inducible system (Vatén et al., 2011) was also employed to block symplastic communication to and from the endodermis. Aberrant periclinal cell divisions and altered endodermal identity in the resulting supernumerary cell files were observed. PIN2, for instance, which is not normally expressed in this cell type, appeared, and it additionally displayed an apolar localisation (Wu et al., 2016). This was consistent with signals moving cell-to-cell being required for correct patterning of this tissue (see, for example, Nakajima et al., 2001). However, in association with these phenotypes, the induced seedlings no longer properly responded to gravitropism. The DR5 signal in the root was altered and auxin did not seem to redistribute correctly upon the trophic stimulus (Wu et al., 2016), a process required for the differential growth and bending (Band et al., 2012). Perturbed active auxin transport (possibly due to the PIN2 appearance) is the likely cause, but a contribution from passive PD mechanisms can not be ruled out.

### Evolutionary perspective on auxin movement

Some authors entertained the idea that, in the context of auxin movement, a passive PD transport system might have *functionally* pre-dated an active one (for example Benítez et al., 2018). Albeit the hypothesis is possible, it might erroneously rely on assumptions of lower ‘complexity’ of the former system.

**Fig. 3.**
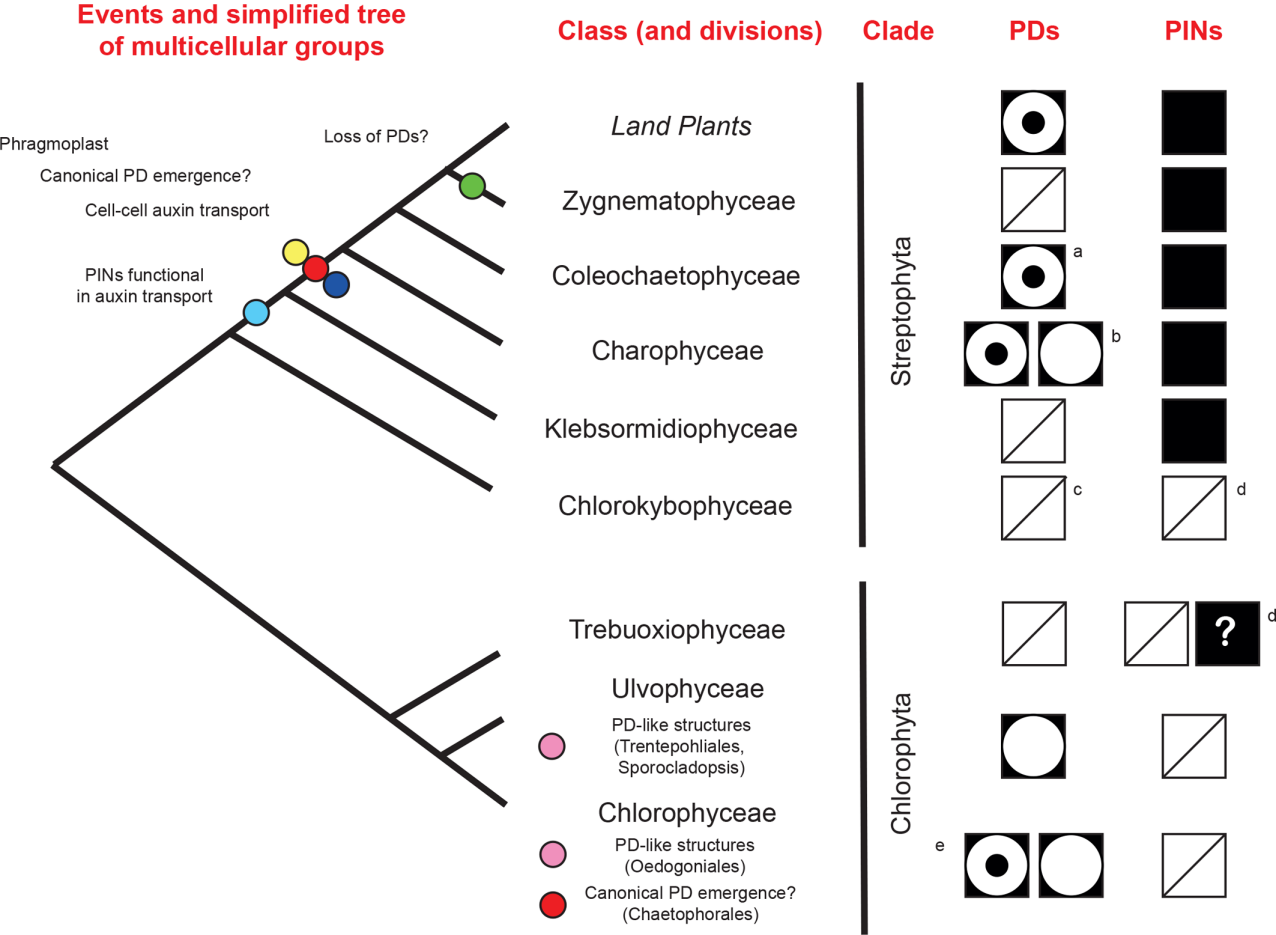
**Evolution of Plasmodesmata and PIN proteins in multicellular groups of the green lineage**. Only classes with multicellular species are depicted in the phylogenetic tree. Branch length does not reflect true evolutionary time. The phylogeny is based on One Thousand Plant Transcriptomes Initiative (2019) and the reviews Fang et al. (2017); Leliaert et al. (2012). The positions of events of interest are suggested on the tree as coloured dots. Presence or absence of PD and PINs in the phylogenetic classes is based on reviews from Nicolas et al. (2018); Raven (2018); Bennett (2015); the paper from De Smet et al. (2011) plus the additional notes. Presence of PD with desmotubules is depicted as a black square with a inscribed white circle and a second internal black circle. PD/PD-like structures without desmotubules are shown as a black squares with empty white inscribed circles. Presence of PINs is shown as a black square. Absence of either structure/protein is shown as a white square with a diagonal line. Notes: (A) no obvious clear image seems available in the literature but the morphology is inferred from Marchant and Pickett-Heaps (1973); (B) controversy regarding the presence (Kwiatkowska and Maszewski, 1986) or absence (Franceschi et al., 1994) of the desmotubule. It is present in at least some contexts (Cook et al., 1997); (C) absence of PD is based on Lokhorst et al. (1988) and Rogers et al. (1980); (D) absence of PINs in Chlorokybophyceae according to Wang et al. (2020), in this study the sequence for a PIN protein was potentially found in Chlorophyta, unlike what concluded in De Smet et al. (2011); (E) desmotubule is present in at least one species based on the figures in Stewart et al. (1973).

Phylogenetic data, when focusing on multicellular families in the green lineage, do not particularly support this notion either. Active, PIN-driven, auxin transport emerged early in the evolution of streptophytes (the clade of land plants) and a *PIN* homolog was described in the filamentous algae *Klebsormidium flaccidum* (order Klebsormidiales). In heterologous systems, the protein performed true auxin transport. However, its localisation in algal cells was not polar and auxin seemed to be released in the environment rather than moving cell-to-cell (Skokan et al., 2019). True polar cell–cell auxin movement seems to appear in the order Charales (Boot et al., 2012). Overall, based on sequence data, PIN proteins are present in all strephophytes (reviewed in Bennett, 2015) but not in more distant chlorophytes (De Smet et al., 2011) (Fig. 3).

The evolution of PD is more complicated and less studied. For instance, it has not been revisited since the sister group to land plants was changed from the order Charales to that of Zygnematales (One Thousand Plant Transcriptomes Initiative, 2019 for the current phylogeny and Zhong et al., 2015 for a review of the changes). Canonical PD forms, with a desmotubule, are restricted to land plants, the order Charales, most likely the order Coleochetales and a specific order in the Chlorophyceae class (Marchant and Pickett-Heaps, 1973 and reviewed in Nicolas et al., 2018; Raven, 2018). PD forms without ER are reported in other orders of the Chlorophyceae and Ulvophyceae classes (reviewed in Nicolas et al., 2018; Raven, 2018). Therefore, in the green lineage, PD and PD-like connections likely appeared multiple times, at least once in streptophytes and multiple times in the Chlorophytes. The development of analogous structures across independent lineages likely bears testimony to the effectiveness of cytosolic continuity as a strategy (among others) for cell–cell communication. Interestingly, PD seem absent in Klabsormidales and Zygnematales (reviewed in Raven, 2018). The former might be a case of functional loss if we assume that PD containing ER evolved after the appearance of the phragmoplast in the green lineage (reviewed in Graham et al., 2000) (Fig. 3). This structure is involved in cell plate formation and ER strands remain embedded inside it (Hepler, 1982). Presence of the desmotubules in some members of the Chlorophyceae class (Stewart et al., 1973) is surprising because those cells do not display a phragmoplast.

In summary, in some charophytes, PD-like structures have been present in absence of PINs but in streptophytes canonical PD likely appeared after PIN emergence and around the same time as these proteins might have started to perform cell–cell auxin movement. Functional specificity between passive and active transport is therefore a more likely explanation for the presence of both systems.

The moss *Physcomitrella patens* (Bryophytes) provides an example of this. While active auxin transport is necessary for apex maintenance, leaf development in the gametophyte phase and for branching regulation in the sporophyte generation of this species (Viaene et al., 2014; Bennett et al., 2014b; Fujita et al., 2008), it is not involved in the control of gametophyte branching (Coudert et al., 2015). Chemical inhibition of PINs or ABCBs transporters did not cause phenotypes. Bidirectional auxin transport, required in the authors’ computational model to reproduce branching patterns, might be instead provided by PD. Treatment with a callose synthesis inhibitor indeed reduced branching in the moss, consistent with higher auxin fluxes in the model. Homologues of callose synthases and glucanases (and presence of callose itself) occur in bryophytes and even basal streptophytes (Del Bem and Vincentz, 2010; Zaveska-Drabkova and Honys, 2017; Gaudioso-Pedraza and Benitez-Alfonso, 2014; Scherp et al., 2001). However, PD regulation by callose has only been shown in vascular plants.

## CONCLUSION

A growing body of publications is now highlighting the functional contribution of auxin moving via PD and the influence on auxin processes of other signals also moving via PD. It is therefore an exciting time for researchers in both fields. Knowledge exchange and synergies between the two communities will be beneficial to push forward some of these aspects. For instance, it will be highly interesting to re-evaluate known processes where auxin levels play key roles and try to investigate if fluxes via PD meaningfully contribute. A few of those cases have been mentioned in this review. Further dissecting the feedbacks and reciprocal impacts of transporter and PD fluxes will also be highly valuable. Manipulating PD aperture in active transport mutants (or manipulating transporters in inducible lines with altered PD aperture) would be challenging yet attractive possibilities. Ultimately, I envisage that comprehensive and quantitative models incorporating the various routes and regulators of auxin movement will be produced for various tissues. These will provide more holistic visions of cells and their robust mechanisms for growth and development. As a community of (molecular) scientific explorers, in coming years, we shall map the symplastic route of auxin movement. While hidden in plain sight, knowledge treasures might await us there.
